# Case of the Passage of an Iron Rod through the Head

**Published:** 1849-02

**Authors:** 


*Case of the Passage of an Iron Rod Through the Head.*—The editor of the Boston Med. and Surg. Journal states, that a note, dated Jan. 3d, from the medical attendant of the man who had an iron rod shot through his head, (see last number of this Journal,) reports the patient to be "walking about the house, and riding out, improving both mentally and physically."

*Quere.* Is there any ground to suspect that the medical attendant may have deceived himself as to the passage of the rod *through* the head? If not, the case is certainly an extraordinary one, and possesses much interest in its relations to surgery, physiology, and psychology.

